# Analytical validation of CanAssist-Breast: an immunohistochemistry based prognostic test for hormone receptor positive breast cancer patients

**DOI:** 10.1186/s12885-019-5443-5

**Published:** 2019-03-20

**Authors:** Arun Kumar Attuluri, Chandra Prakash V. Serkad, Aparna Gunda, Charusheila Ramkumar, Chetana Basavaraj, Ljubomir Buturovic, Lekshmi Madhav, Nirupama Naidu, Naveen Krishnamurthy, R. Prathima, Suchita Kanaldekar, Manjiri M. Bakre

**Affiliations:** 1 OncoStem Diagnostics Pvt Ltd, # 4 Raja Ram Mohan Roy Rd, Aanand Tower, 2nd Floor, Bangalore, 5600027 India; 2 Clinical Persona Inc, 932 Mouton Circle, East Palo Alto, CA 94303 USA

**Keywords:** CanAssist-breast, Reproducibility, Repeatability, Analytical validation, Immunohistochemistry

## Abstract

**Background:**

CanAssist-Breast is an immunohistochemistry based test that predicts risk of distant recurrence in early-stage hormone receptor positive breast cancer patients within first five years of diagnosis. Immunohistochemistry gradings for 5 biomarkers (CD44, ABCC4, ABCC11, N-Cadherin and pan-Cadherins) and 3 clinical parameters (tumor size, tumor grade and node status) of 298 patient cohort were used to develop a machine learning based statistical algorithm. The algorithm generates a risk score based on which patients are stratified into two groups, low- or high-risk for recurrence. The aim of the current study is to demonstrate the analytical performance with respect to repeatability and reproducibility of CanAssist-Breast.

**Methods:**

All potential sources of variation in CanAssist-Breast testing involving operator, run and observer that could affect the immunohistochemistry performance were tested using appropriate statistical analysis methods for each of the CanAssist-Breast biomarkers using a total 309 samples. The cumulative effect of these variations in the immunohistochemistry gradings on the generation of CanAssist-Breast risk score and risk category were also evaluated. Intra-class Correlation Coefficient, Bland Altman plots and pair-wise agreement were performed to establish concordance on IHC gradings, risk score and risk categorization respectively.

**Results:**

CanAssist-Breast test exhibited high levels of concordance on immunohistochemistry gradings for all biomarkers with Intra-class Correlation Coefficient of ≥0.75 across all reproducibility and repeatability experiments. Bland-Altman plots demonstrated that agreement on risk scores between the comparators was within acceptable limits. We also observed > 90% agreement on risk categorization (low- or high-risk) across all variables tested.

**Conclusions:**

The extensive analytical validation data for the CanAssist-Breast test, evaluating immunohistochemistry performance, risk score generation and risk categorization showed excellent agreement across variables, demonstrating that the test is robust.

**Electronic supplementary material:**

The online version of this article (10.1186/s12885-019-5443-5) contains supplementary material, which is available to authorized users.

## Background

Only 15% of patients with early-stage breast cancer benefit from adjuvant chemotherapy [1–4]. Identification of these patients who are at high risk for distant recurrence and hence would benefit from adjuvant chemotherapy is intricate and depends upon the tumor biology, clinical and pathological parameters.

Most tests that are currently available to predict risk of distant recurrence for breast cancer utilize quantitative gene expression-based platforms [5–8]. These tests are expensive and require specialized equipment in the laboratory. We have developed and validated CanAssist-Breast (CAB) test that uses a cost-effective, gold standard methodology of immunohistochemistry (IHC) along with key clinicopathological factors to determine risk of distant recurrence in early stage HR+ breast cancer [9].

CAB uses IHC based evaluation of expression levels of 5 key biomarkers (CD44, N-cadherin, pan-cadherin, ABCC4 and ABCC11) and three clinicopathological prognostic parameters tumor size, tumor grade and node status (as obtained from the medical records from hospitals where these patients were treated) to arrive at a “CAB-Risk Score”. “CAB Risk Score” classifies patients into two categories, either low- or high-risk for distant recurrence. The detailed rationale for choosing these biomarkers is explained in the prior publication describing the development of CAB test [9]. Briefly, these five biomarkers play a pivotal role in cancer progression, drug-resistance leading to cancer recurrence. CD44 is a stem cell marker, cadherins contribute to epithelial-mesenchymal transition (EMT), while ABCC4 and ABCC11 are ATP binding cassette transporter proteins involved in drug efflux leading to drug resistance. “CAB Risk Score” classifies patients into two categories, either low- or high-risk for distant recurrence.

**Fig. 1:**
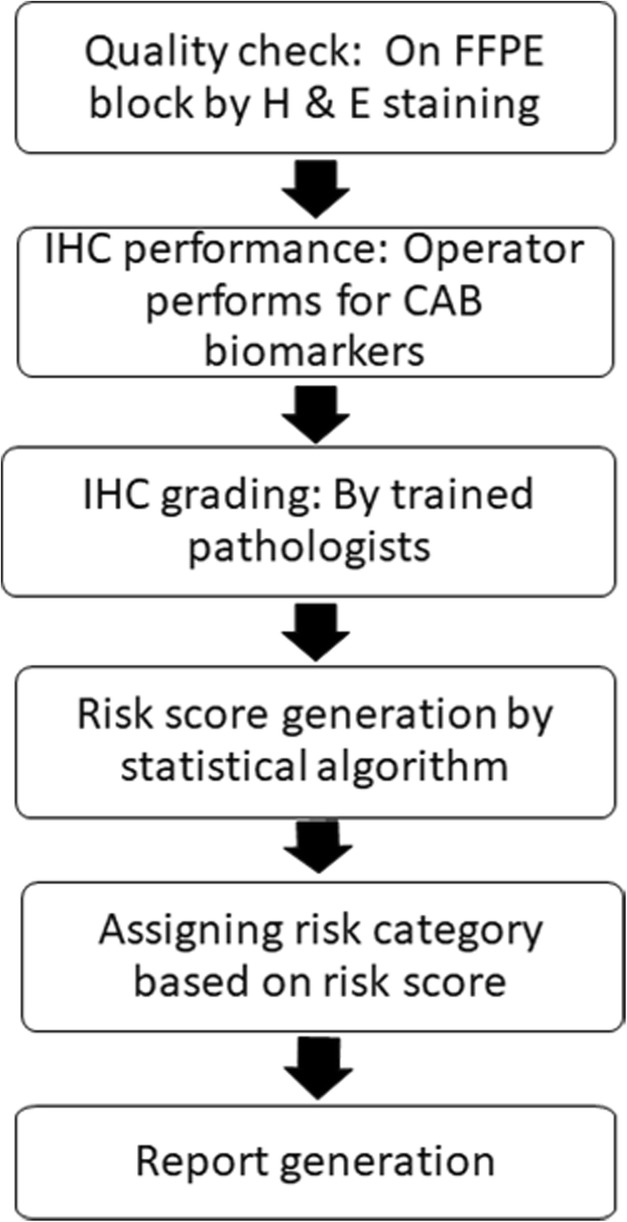
CanAssist-Breast (CAB) test work flow: The flow chart depicts various steps involved in the whole process of testing a tumor sample for CanAssist-Beast. The FFPE tumor block undergoes a quality check by H&E staining. IHC is performed for all 5 CAB biomarkers. Trained observer (pathologist) grades the slides. The statistical algorithm generates risk-score using gradings of biomarkers and clinical parameters. Based on risk score, risk category is assigned and finally report is generated

The steps involved in the CAB test are described in Fig. 1. It includes an initial sample quality check of the tumor sample by Haemotoxylin and Eosin (H&E) staining, followed by IHC staining for CAB biomarkers. A machine learning based statistical algorithm uses the IHC gradings of CAB biomarkers along with the patient’s clinical parameters to generate a risk score ranging between 0 and 100 with a cut-off at 15.5. Patients with a risk score below the cut-off are considered to `low-riskʼ of recurrence and patients with risk score above the cut-off are considered to have `high-riskʼ of recurrence.

**Fig. 2:**
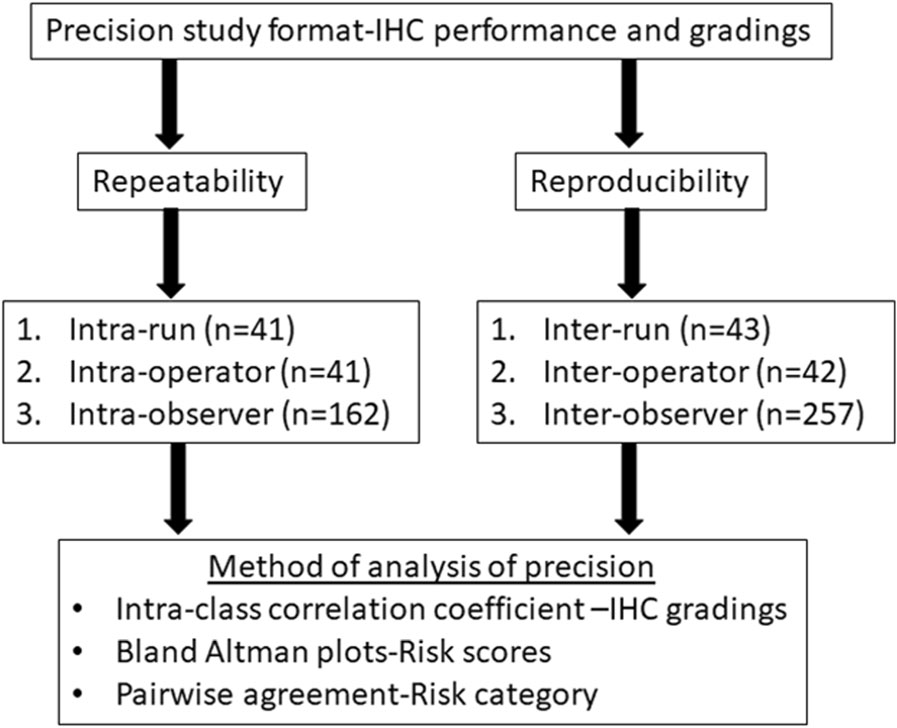
Overview of Precision study for analytical validation of CAB test: All potential sources of variation in the CAB test have been shown along with the methodology used to analyze. Sample size for each of the variable tested is given in brackets

The Evaluation of Genomic Applications in Practice and Prevention Working Group (EGAPP) has postulated recommendations for validation of gene expression-based prognostic and predictive tests in breast cancer that includes both clinical and analytical components [10]. Several factors influence the IHC testing process that may have a bearing on the ultimate result and its interpretation [11]. The College of American Pathologists (CAP) and Clinical Laboratory Standards Institute (CLSI) have laid down guidelines for analytical validation of IHC based tests [12, 13]. Analytical validation is essential to demonstrate the repeatability and reproducibility of a test across variables that affect its’ performance. Repeatability demonstrates precision among repeated measurements taken under the same conditions, such as in the same run or performed by the same operator. Reproducibility demonstrates precision for measurements taken under different conditions like multiple -operators, laboratories and observers. As per CLSI guidelines, precision for a centralized laboratory developed test like CanAssist-Breast should encompass variations due to runs, operators and pathologists/observers. This study has been carried out based on all these recommendations to assess the repeatability and reproducibility of the CAB test using a total of 309 samples as shown in Fig. 2.

Since the CAB risk score generation depends on the cumulative gradings of five different biomarkers, it is important that we evaluate the effect of variability in the gradings of individual biomarkers on risk scores and risk category assignments. To address this, precision analysis was further extended to the CAB risk score generation and risk categorization.

Thus, analytical validation study described here aims to establish repeatability and reproducibility of the CAB test and is performed in conjunction with CAP recommendations and CLSI guidelines:

1. To analyze the effect of factors influencing IHC performance and gradings
2. To assess the effect of the factors on CAB risk score generation and risk categorization

## Methods

### Tumor sample selection

Tumor samples were obtained from patients diagnosed with invasive breast carcinoma. The sample selection criteria for the current analytical performance study were similar to that used for the development of CanAssist-Breast [9]. Furthermore, only those blocks that had more than 30% tumor content as per H & E staining were selected for this study to ensure the availability of sufficient tumor tissue for testing multiple markers and for performing repeatability and reproducibility experiments. The patients included in the study were HR+ and HER2-. These patients had tumors which were either N0 (node negative) or N1 (up to 3 positive lymph nodes) and with tumor size of < 2 cm (T1) or 2-5 cm (T2). We relied on the hospital for information on tumor size, node status and grade. Out of a total of 309 breast cancer samples used for the study, more than 90% of samples were of disease Stage 1 and 2 and were moderately (Nottingham grade 2) or poorly differentiated (Nottingham grade 3). We followed a nested approach in which the same tumor samples were used across various experiments.

### Assessment and processing of FFPE blocks

The FFPE blocks were physically examined for proper labelling. Integrity of block was assessed by a senior technician for any kind of processing artifacts. The blocks were sectioned into 3-μm sections for H & E staining and IHC experiments. The H & E stained slides were assessed for tumor content and tissue artifacts like necrosis and hemorrhages. The blocks with tissues of necrosis/hemorrhage greater than 10% were rejected. IHCs were performed on tissue slides within a week of sectioning.

### Immunohistochemical study

According to CAP and CLSI guidelines, optimal antibody concentration and antigen retrieval conditions were established as integral components of standardizing IHC based assays. Immunohistochemistry was performed on five serial sections of the FFPE tumor blocks for the 5 CAB biomarkers. Positive and negative controls were included in every IHC experiment. The detailed IHC staining protocol for the 5 CAB biomarkers has been performed as described previously [9]. The details of antibody specifications and IHC performance is mentioned in ‘Additional file 1’ while staining location and controls used are mentioned below.

#### Positive and negative controls

Positive control used is a previously tested breast cancer tissue sample that expressed high levels (> 60% staining) of the biomarkers. Either an isotype matched non-specific IgG, instead of the specific antibody or negative reagent control were used as negative controls. All IHC slides were graded by qualified Pathologists who have been trained to grade these slides in our central laboratory where all tests are performed.

#### IHC grading

We did not assign a specific cut-off value for assessing positivity of IHC staining for any of the five biomarkers. Each biomarker was graded as absolute value of percentage of the tumor cells stained at a specific location (membrane or cytoplasmic) on a continuous scale of 0–100 and based on the intensity of the staining on a scale of 0–3 [9]. CD44, ABCC4, ABCC11 were graded on the cell membrane and N-cadherin and pan-cadherin were graded on the cytoplasm. These absolute values of percentage of staining and intensity of staining along with clinical parameters (tumor size, grade and node status) were used by the statistical algorithm to generate a risk score.

### Precision experiments

To establish precision of CAB test (repeatability and reproducibility), variations due to operator, run and observer (pathologist) (Fig. 2) were evaluated using an appropriately powered sample number. This was established by statistical calculations to estimate probability of success (PoS) of agreement for each parameter. The acceptability criteria for the lower limit of the 95% CI was set at 80% and the sample size number corresponding to PoS of 70% was chosen.

**Fig. 3:**
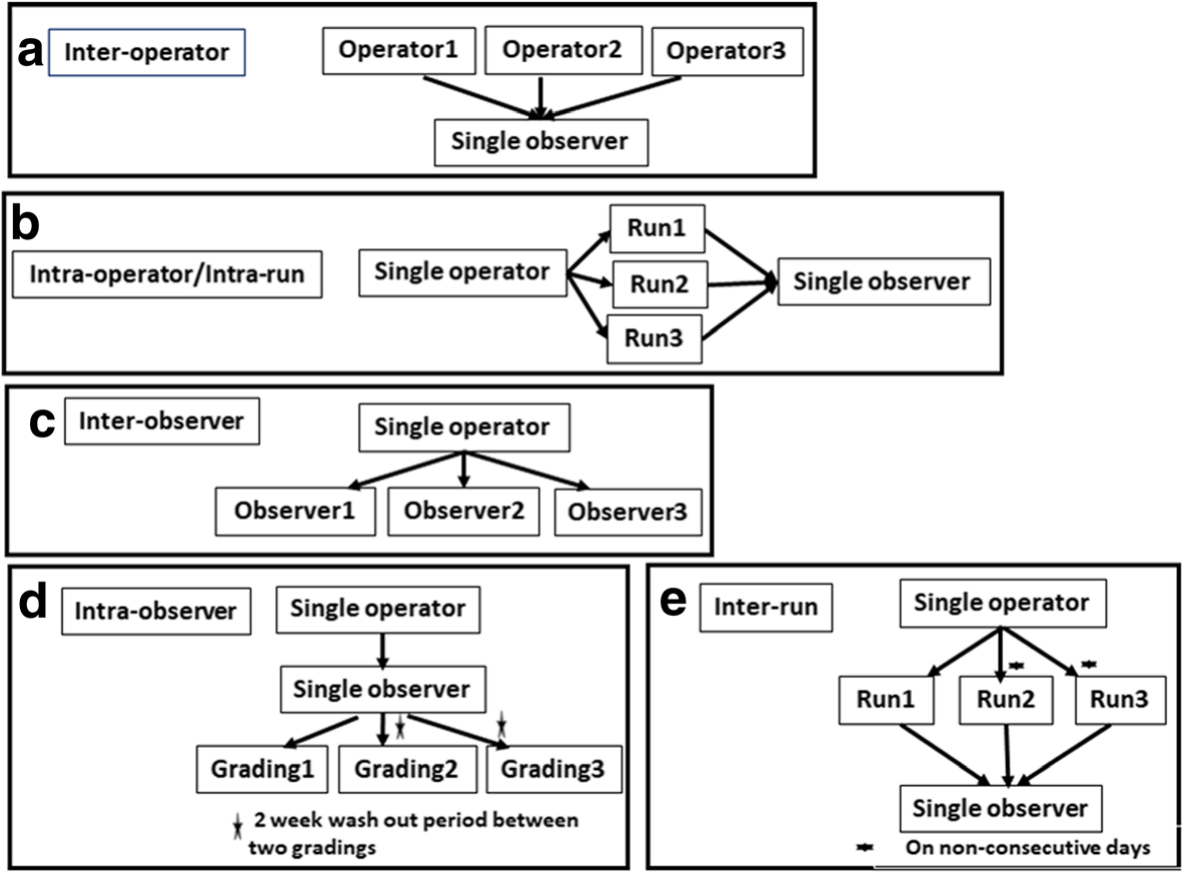
Design of Precision experiments Potential sources of variations are tested as detailed above (**a**-**e**) with sample size of 309, samples ranged from 41 to 257 for various experiments performed

The experimental design for investigating the various sources of variabilities is shown in Fig. 3. Three operators performed IHC experiments for all CAB biomarkers and grading was performed by a single observer to assess inter-operator variation (Fig. 3a). Intra-operator/intra-run variability was evaluated by a single operator performing IHC on the same sample in triplicates on the same day and all the three replicates being graded by a single observer (Fig. 3b). Inter-observer variation was assessed with a single operator performing the IHC experiments for all CAB biomarkers and graded independently by three qualified observers (Fig. 3c). Intra-observer variation was assessed with a single operator performing the IHC experiment followed by gradings by the same observer three times, with a two week washout period between repeat gradings (Fig. 3d). Inter-run variation was evaluated by the same operator performing repeat IHC experiments on three non-consecutive days and grading for all these repeats being done by a single observer (Fig. 3e).

### Statistical analyses

#### Precision analysis on IHC gradings

This was assessed using Intra-class Correlation Co-efficient (ICC) using the MedCalc software. Correlation co-efficients were measured for IHC gradings for each of the 5 biomarkers. Comparisons were made on the grading by each observer with the average grading of 3 observers (inter-observer); each grading by an observer with average of triplicate gradings by the same observer (intra-observer); grading by a single observer for each IHC experiment with the average of gradings from triplicate IHC experiments done by the same operator (intra-operator/intra-run); grading by a single observer for each IHC experiment performed by an operator compared to the average gradings of the independent IHC experiments performed by 3 operators (inter-operator); each grading by the same observer from IHC experiments done by the an operator with the average gradings of IHC experiments done on three consecutive days by the same operator (inter-run). Thus, three ICC values were obtained for every category. The final ICC values were expressed as an average of these three ICC values. ICC values below 0.4 indicate poor agreement; between 0.4 and 0.59 indicate fair to moderate agreement; between 0.6 and 0.74 indicated good agreement and greater than 0.75 indicated almost excellent agreement [14].

#### Precision on risk-scores

Bland Altman plots were used to assess the agreement on risk scores across various variables tested. For all comparisons done in this analytical study, individual reading/score is correlated to average readings/scores. Risk scores computed from the gradings of the 5 CAB biomarkers done by one observer was compared to average risk score obtained by gradings from three observers (inter-observer) or three gradings by same observer (intra-observer) or gradings obtained by a single observer on IHC performed by three different operators (inter-operator) or gradings obtained by triplicate IHC experiments done by the same operator and graded by a single observer (intra-operator/intra-run) or gradings done by the same observer on IHC performed by a single operator on three non-consecutive days (inter-run). In these plots, presence of samples between the two dotted lines (mean + 2SD) was considered to have good agreement between two measurements that were analyzed [15].

#### Precision on risk-categorization

Pairwise agreement was calculated using the majority call/consensus method as described at http://onlinelibrary.wiley.com/doi/10.1111/ajt.12193/full. Agreement was calculated as per the CLSI guidelines. In this method, the consensus prediction between 2 out of 3 observers was considered paramount (for eg: Obs1- Low-risk, Obs2- High-risk, Obs3- Low-risk, Majority call- Low-risk), and each individual observers’ prediction was compared to the consensus prediction and pairwise agreement was assessed. Statistical analysis for inter-observer variation was performed by calculating Overall Percent Agreement (OPA, agreement between both low- and high-risk predictions) Negative Percent Agreement (NPA, agreement between low-risk predictions) and Positive Percent Agreement (PPA, agreement between high-risk predictions) as described in the CLSI guidelines [13]. 95% confidence intervals were calculated using Boot-Strap method.

## Results

### Expression levels of CAB biomarkers

**Fig. 4:**
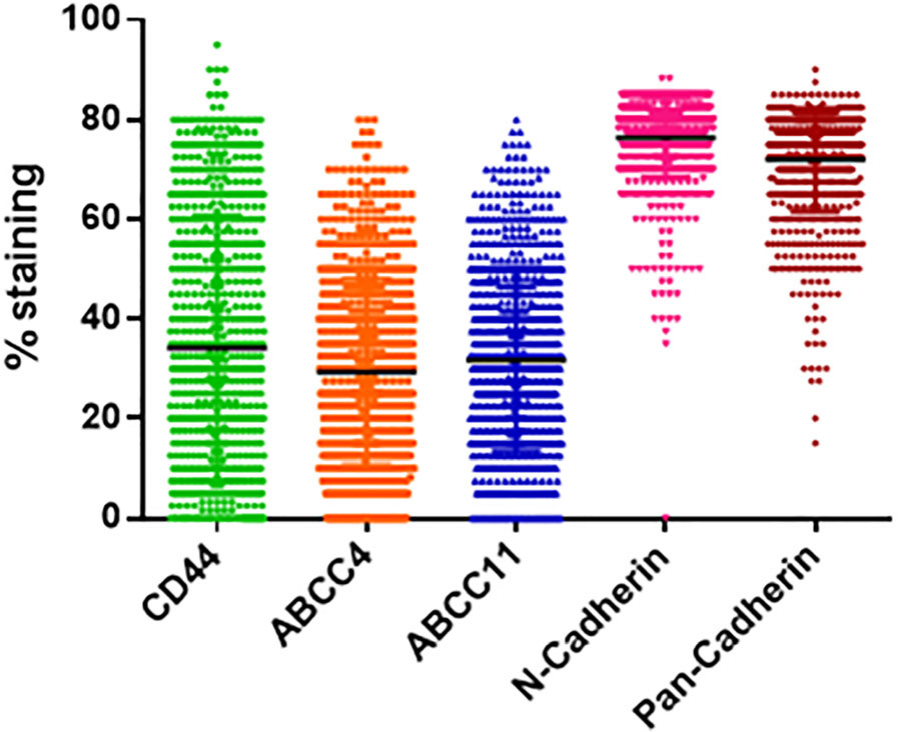
Spread of Biomarker staining Dot plot shows the spread of the staining percentage for the five biomarkers

All the five biomarkers showed a range of expression across tumor samples in our patient cohort (Fig. 4). The typical IHC staining pattern for the individual biomarkers is shown in Additional file 2: Figure S1. The dot plot in Fig. 4 shows that the expression levels of membrane stained biomarkers CD44, ABCC4 and ABCC11 span a wider range from 0 to 100%, while the cytoplasmic biomarkers, N-Cadherin and pan-Cadherins primarily have higher expression levels (Fig. 4). Very few patient samples had N-Cadherin and pan-Cadherin expression levels of less than 20% (Fig. 4).

### Analysis of parameters contributing to CanAssist-Breast variability

#### Inter-observer (pathologist) variability

The quantification of IHC results is dependent on the subjective evaluation of percentage and intensity of staining even by trained observers [16, 17]. Variation between gradings for the same biomarker by any two observers (inter-observer) is considered the most important variable in IHC [18, 19]. For all biomarkers, ICC among three observers on a sample size of 257 cases was ≥0.75 (Table 1, row 1), and demonstrated a strong agreement between observers with respect to IHC gradings of each biomarker.

**Table 1:** Correlation between IHC gradings of 5 biomarkers across variables: Intra-class correlation coefficient (ICC) for IHC gradings of all 5 CAB biomarkers tested for inter-observer, intra-observer, inter-operator, inter-run and intra-run variability

Variables	Percentage of cells stained					Intensity of staining
	CD44	ABCC4	ABCC11	N-Cadherin	Pan-Cadherin	Pan-Cadherin
Inter-observer	0.98	0.84	0.83	0.79	0.75	0.84
Intra-observer	0.96	0.91	0.91	0.92	0.92	0.81
Inter-operator	0.98	0.97	0.97	0.86	0.90	0.94
Inter-run	0.97	0.93	0.97	0.97	0.95	0.93
Intra-run/Intra-operator	0.99	0.99	0.99	0.99	0.98	0.97

Place holder for Table 1 Can be placed in Inter-observer (pathologist) variability section in “Results” at line no. 221

#### Intra-observer (pathologist) variability

Intra-observer variation was assessed on a sample size of 162 for each of the 5 biomarkers. Triplicate gradings for the same slide by a single observer with a washout period of two weeks between 2 gradings showed strong agreement across all the 5 biomarkers, with ICC of ≥0.8 (Table 1, row 2).

#### Inter-operator variability

Inter-operator variation is an important confounding factor in the performance of an IHC based test. To study inter-operator variation, 3 operators performed the CAB test on the same FFPE blocks of different patients (*n* = 42), and the resulting slides were graded by a single observer. Strong agreement was indicated across all the 5 biomarkers for three operators with an ICC of ≥0.85 (Table 1, row 3).

#### Inter/Intra-run variability

CAP guidelines postulate that demonstration of inter-run in at least 10 samples is essential to determine repeatability of IHC staining [13]. We performed inter-run variation analysis using 43 samples as described in methods. The ICC for each CAB marker across runs was ≥0.93 (Table 1, row 4) signifying a strong agreement.

Intra-run/intra-operator variation was assessed as a measure of repeatability by running triplicates within a single run (*n* = 41). There was a strong agreement for each of the CAB biomarkers within runs with an ICC of ≥0.97 (Table 1, row 5).

### Precision on generation of risk-scores

**Fig. 5:**
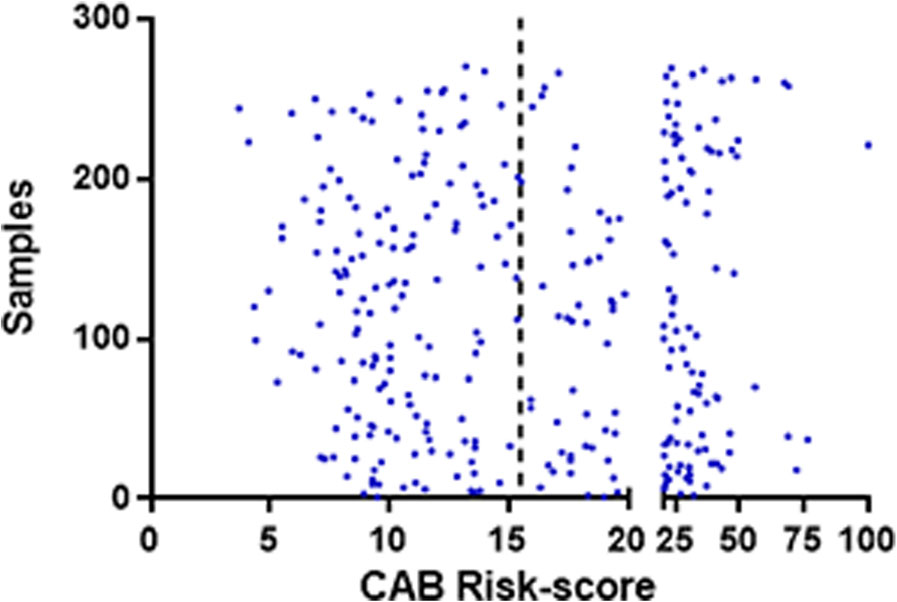
Distribution of CAB risk scores across all the tumor samples used for precision experiments: Dot plot shows the distribution of risk scores for all the tumor samples (*n* = 309) used for the precision experiments.

The tumor samples (*n* = 309) used in this study had risk scores distributed across the whole range of CAB test from 0 to 100 (Fig. 5). Around 20–30% of samples had risk scores around clinical decision point (15.5 ± 2.5 or CAB score:13–18) for all the variables tested.

**Fig. 6:**
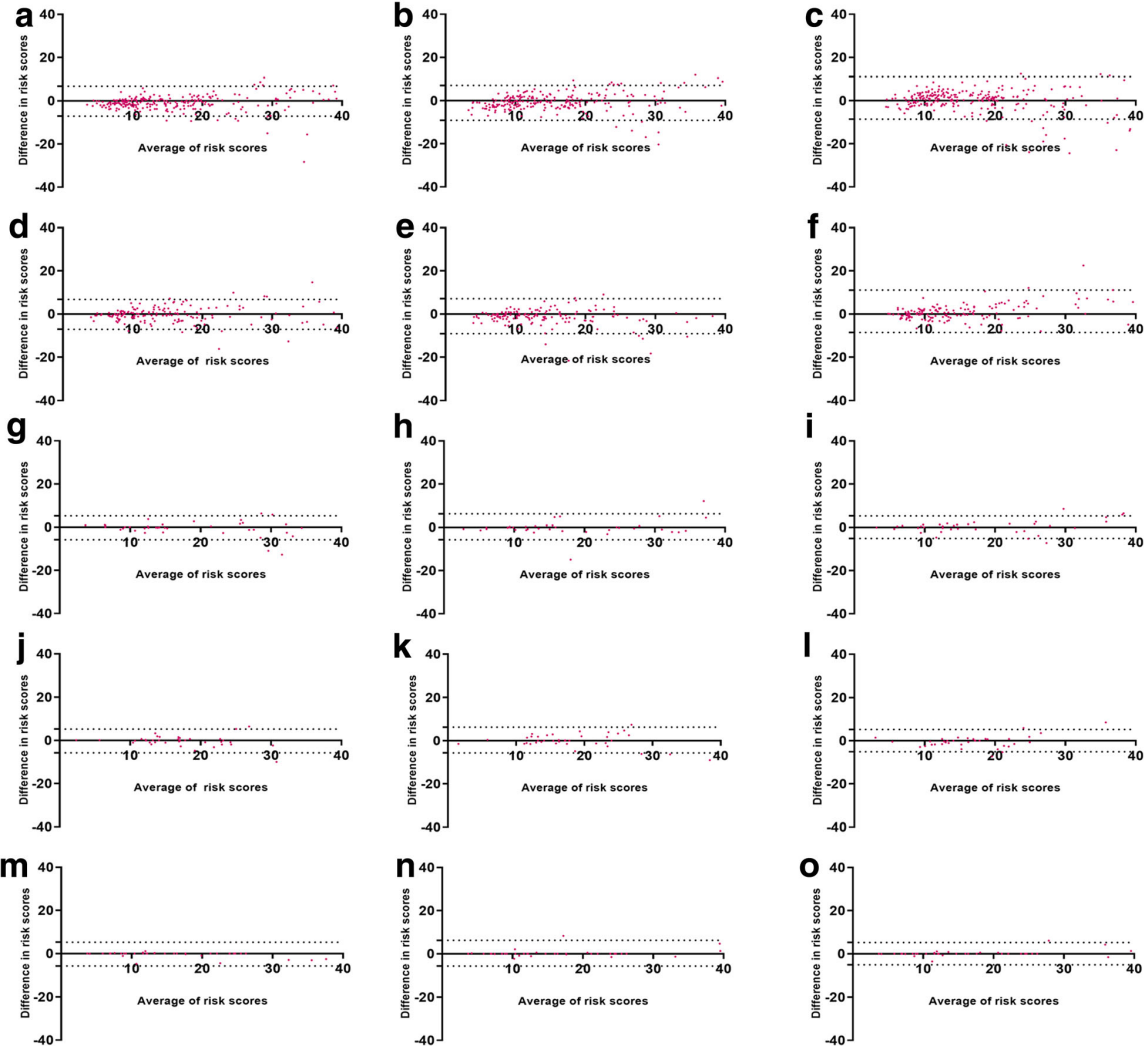
Bland Altman Plots showing correlation for risk scores between variables: Bland Altman plot for risk score between average scores of three observers versus observer 1 (**a**),observer 2 (**b**), observer 3 (**c**); average scores of three gradings of single observer versus grading 1 (**d**), grading 2 (**e**), grading 3 (**f**); average scores of three gradings of 3 operators versus operator 1 (**g**), operator 2 (h), operator 3 (**i**); average scores of three runs performed by an operators versus run 1 (**j**), run 2 (k), run 3 (**l**); average scores of three readings performed by an operator in a run versus reading 1 (**m**), reading 2 (**n**), reading 3 (**o**)

Pair-wise agreement on risk scores was assessed using Bland-Altman plots to check for the effect of IHC grading on prediction of risk-scores [15]. We established the agreement between each of the variable’s risk score to that of the average risk score as described in methods. The schematic representation of the testing method for this analysis is included in Additional file 3: Figure S2. Bland-Altman plot demonstrated that variability in the risk scores for inter-observer (Fig. 6, a-c), intra-observer (Fig. 6, d-f), inter-operator (Fig. 6, g-i), inter-run (Fig. 6, j-l) and intra-run/intra-operator (Fig. 6, m-o), were within acceptable limits.

**Fig. 7:**
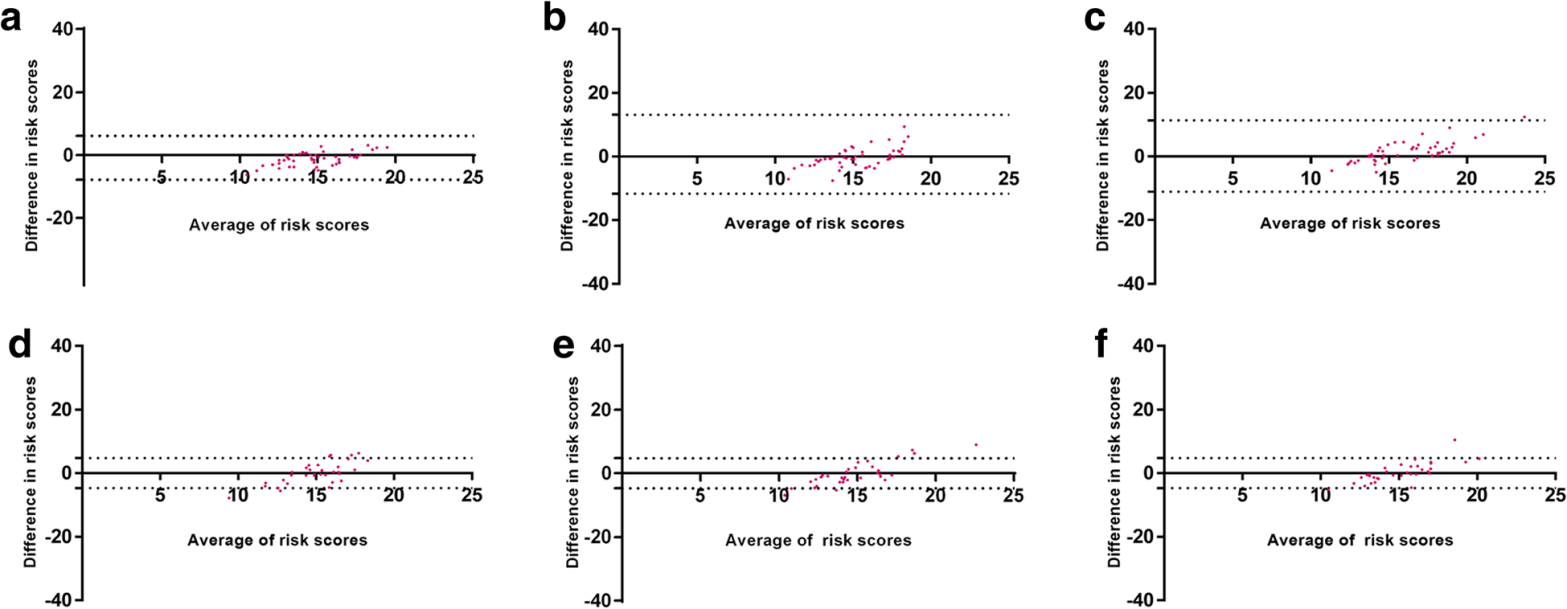
Bland Altman Plots for risk scores between variables around CAB clinical decision point, 13–18: Bland Altman plots for risk scores between average scores of three observers versus observer 1 (**a**), observer 2 (**b**), observer 3 (**c**); average scores of three gradings of single observer versus grading 1 (**d**), grading 2 (**e**), grading 3 (**f**)

As the clinical decision point determines the risk category and could determine treatment decisions for the patient, it is important that the test exhibits tantamount reproducibility around the threshold point. We analyzed inter- (*n* = 50) and intra- (*n* = 33) observer variability using Bland Altman plots for samples with risk scores around the clinical decision point (CAB scores of 15.5 ± 2.5). The data showed strong agreement between inter- (Fig. 7, a-c) as well as intra- (Fig. 7, d-f) observer variability around the clinical decision point.

### Precision on risk category prediction

After determining high concordance in IHC gradings and risk scores between various variables tested, we assessed agreement across observers, operators and runs on risk categorization by majority call or consensus method as described in methods. Results of all the variabilities analyzed showed ≥90% agreement for low-risk (NPA), high-risk (PPA) and over-all (OPA) predictions with tight 95% confidence intervals (Table 2).

**Table 2:** Correlation between risk categories across variables: Negative Percent Agreement (NPA), Positive Percent Agreement (PPA) and Overall Percent Agreement (OPA) for all the variables tested and the 95% CI scores are provided

Parameters	OPA (95% CI)	NPA (95% CI)	PPA (95% CI)
Inter-observer	93.3 (91.2, 94.9)	93.1 (90.3, 95.4)	93.4 (90.2, 95.8)
Intra-observer	91.3 (88.4, 93.7)	91.7 (87.9, 94.6)	90.8 (85.8, 94.4)
Inter-operator	95.2 (89.9, 98.2)	93.3 (83.8, 98.2)	97.0 (89.5, 99.6)
Inter-run	95.3 (90.2, 98.3)	93.8 (82.8, 98.7)	96.3 (89.6, 99.2)
Intra-run/intra-operator	99.2 (95.6, 100.0)	98.3 (91.1, 100.0)	100.0 (94.3, 100.0)

OPA: overall percent agreement, NPA: Negative percent agreement, PPA: Positive percent agreement, CI: confidence Interval

Place holder for Table 2 can be placed in ‘Precision on risk-category prediction’ in “Results” section at line no. 266

## Discussion

CanAssist-Breast is a laboratory developed test that uses a well-accepted technology, IHC along with a complex algorithm to predict risk of cancer recurrence. Demonstration of the analytical performance is a critical requirement for all the diagnostic tests to ensure that accurate results are reported for every patient. Clinical utility of a test not only requires that the test is clinically validated but also is analytically validated for its precision, repeatability and reproducibility. EGAPP has reported that many of the tests lack either analytical validation or corresponding available data is insufficient [10]. Experiments were designed to prove that the CAB test is reproducible and repeatable across various variables, both technical and subjective. The data presented in this study demonstrates robustness of the test factoring in all potential sources of variability.

One of the challenges inherent in demonstrating the analytical validity of any IHC based test is the lack of comprehensive evaluation criteria and a gold standard of reference. To overcome this, we adopted the evaluation criteria set by CAP and CLSI recommendations on thorough analysis of steps involved in validation of new IHC based tests [12, 13]. CLSI has documented detailed procedures for designing repeatability and reproducibility experiments and statistical methodologies for the analytical validation of a test [13].

As CAB test uses five biomarkers using the IHC technique, we evaluated the variations brought in by IHC gradings for each of these five biomarkers using appropriate statistical methodology. There was strong agreement on gradings for all CAB biomarkers for the operator and run variables with ICC of > 0.75 (Table 1) suggesting that performance of IHC was not influenced by change in operator or the day on which the IHC experiment was performed. Strong agreement on risk scores obtained by the cumulative grading of all biomarkers is demonstrated by Bland Altman plots. The intra-run, inter-run and inter-operator plots have data points within the acceptable limits of agreement [15], indicating that robustness at the level of gradings is translated to risk scores. Further pair-wise agreement analysis for risk categories for these run and operator variables specifically inter-operator (OPA, NPA and PPA > 93%), and inter-run (OPA, NPA and PPA > 93%) and intra-run (OPA, NPA and PPA > 98%) parameters demonstrated that these variables do not affect CAB test based risk category prediction (Table 2).

Subjective interpretation/grading of IHC stained slides leading to inter-observer variation is the major drawback of IHC based tests [16, 17]. We have addressed this concern by testing inter-observer variation using a large cohort of a sample size of 257. ICC for the IHC gradings for all the five CAB biomarkers (ranged between 0.75 to 0.98, Table 1) and Bland-Altman plots for CAB risk score generation (Fig. 6) demonstrated strong agreement between observers indicating that the CAB test does not suffer from inter-observer variability with respect to IHC interpretation and risk score generation (Table 2). Pairwise agreement analysis for low-, high- and overall risk predictions had a high concordance of > 90% in observer variation experiments (Table 2). The performance of the CAB test is evaluated at clinical decision point also (Fig. 7). High variation at the clinical decision point directly affects patients’ care by influencing the treatment decisions. Bland Altman plots for inter-observer variation showed high precision thus demonstrating the optimal performance of the test at the clinical decision point.

Although CAB risk score generation uses three clinical parameters along with IHC gradings of five biomarkers, in this study we have not assessed the variations brought in by clinical parameters namely node status, Nottingham grade and tumor size. All the three clinical parameters are taken from the patient’s histopathology report routinely used by the clinicians for planning treatment.

Currently available risk-stratifying multi-gene tests use genomic methods and can be expensive especially in low-resource countries where expenses for the tests and treatment are out of pocket. Use of IHC (which is a gold standard technique) helped us to bring down the cost of CAB test to a fraction of the cost of currently available multi-gene tests, thus making it more affordable even in low-resource settings. Furthermore, the CAB test has been clinically validated using a large cohort of 900 retrospective patient samples in a global, multi-center study [20] which establishes the clinical utility of CAB comparable to other multi-gene tests. Clinical validation of CAB test in a multi-center randomized trial, similar to the other multigene tests available in the market [21–23], would be the next logical approach and such studies are currently underway.

## Conclusion

We have thus conclusively shown that CanAssist-Breast, multi-marker IHC-based test is robust across several repeatability and reproducibility variables tested making it a reliable, cost-effective and accurate prognostic test to stratify patients with early stage HR+ breast cancer into low- or high-risk for distant recurrence.

## Additional files

Additional file 1: Additional information on methods. (DOCX 14 kb)

Additional file 2: **Figure S1.** Representative IHC images of the CanAssist-Breast biomarkers: IHC images of CanAssist-Breast biomarkers captured at 40X magnification a. CD44, b. ABCC4, c. ABCC11, d. N-cadherin, e. Pan-cadherin respectively. (TIF 10398 kb)

Additional file 3: **Figure S2.** Analysis employed for assessing ‘Precision on risk scores’. Schematic representation of the analysis employed in assessing precision on risk scores for all variables tested (a-e). (TIF 347 kb)

## Abbreviations

CAB: CanAssist-Breast
CAP: College of American Pathologists
CLSI: Clinical Laboratory Standards Institute
EGAPP: Evaluation of Genomic Applications in Practice and Prevention Working Group
EMT: Epithelial Mesenchymal Transition
FFPE: Formalin Fixed Paraffin Embedded
H&E: Hematoxylin & Eosin
HR: Hormone Receptor
ICC: Intra class correlation coefficient
IDC: Invasive Ductal Carcinoma
IHC: Immunohistochemistry
PoS: Probability of success

## References

[1] Fisher B, Dignam J, Wolmark N, DeCillis A, Emir B, Wickerham DL (1997). Tamoxifen and chemotherapy for lymph node-negative, estrogen receptor-positive breast cancer. J Natl Cancer Inst.

[2] Fisher B, Jeong J-H, Bryant J, Anderson S, Dignam J (2004). Fisher, et al. treatment of lymph-node-negative, oestrogen-receptor-positive breast cancer: long-term findings from National Surgical Adjuvant Breast and bowel project randomised clinical trials. Lancet.

[3] Fisher B, Costantino J, Redmond C, Poisson R, Bowman D, Couture J (1989). A randomized clinical trial evaluating tamoxifen in the treatment of patients with node-negative breast cancer who have estrogen-receptor-positive tumors. N Engl J Med.

[4] Early Breast Cancer Trialists’ Collaborative Group (EBCTCG) (2005). Effects of chemotherapy and hormonal therapy for early breast cancer on recurrence and 15-year survival: an overview of the randomised trials. Lancet.

[5] van de Vijver MJ, He YD, van’t Veer LJ, Dai H, Hart AAM, Voskuil DW (2002). A gene-expression signature as a predictor of survival in breast cancer. N Engl J Med.

[6] Paik S, Shak S, Tang G, Kim C, Baker J, Cronin M (2004). A multigene assay to predict recurrence of tamoxifen-treated, node-negative breast cancer. N Engl J Med.

[7] Wallden B, Storhoff J, Nielsen T (2015). Development and verification of the PAM50-based Prosigna breast cancer gene signature assay. BMC Med Genet.

[8] Dubsky P, Filipits M, Jakesz R, Rudas M (2013). Endopredict improves the prognostic classification derived from common clinical guidelines in ER-positive, HER2-negative early breast cancer. Ann Oncol.

[9] RamKumar C, Buturovic L, Malpani S (2018). Development of a novel proteomic risk-classifier for prognostication of patients with early-stage hormone receptor-positive breast Cancer. Biomark Insights.

[10] Evaluation of Genomic Applications in Practice and Prevention (EGAPP) Working Group (2009). Recommendations from the EGAPP working group: can tumor gene expression profiling improve outcomes in patients with breast cancer?. Genet Med.

[11] O’Hurley G, Sjorstedt E, Rahman A (2014). Garbage in, garbage out: a critical evaluation of strategies used for validation of immunohistochemical biomarkers. Mol Oncol.

[12] Patrick LF, Linda AB, Lisa AF (2014). Principles of analytic validation of immunohistochemical assays: guideline from the College of American Pathologists Pathology and Laboratory Quality Center. Arch Pathol Lab Med.

[13] Quality Assurance for Design Control and Implementation of IHC; Approved guideline - Second Edition. 2011. CLSI- I/LA28-A2.

[14] Cicchetti and Domenic V (1994). Guidelines, criteria and rules of thumb for evaluating normed and standardized assessment instruments in psychology. Psychol Assess.

[15] Bland M, Altman DG (1986). Statistical methods for assessing agreement between two methods of clinical measurement. Lancet..

[16] Adams EJ, Green JA, Clark AH, Youngson JH. Comparison of different scoring systems for immunohistochemical staining. J Clin Patho 1999; l52: 75–77. 10.1136/jcp.52.1.75 PMC501013 10343618

[17] van Diest PJ, Weger DR, Lindholm J (1996). Reproducibility of subjective immunoscoring of steroid receptors in breast cancer. Anal Quant Cytol Histol.

[18] Kirkegaard T, Edwards J, Tovey S, McGlynn LM (2006). Observer variation in immunohistochemical analysis of protein expression, time for a change?. Histopathology..

[19] Maia DM (1999). Immunohistochemical assays for HER2 overexpression. J Clin Oncol.

[20] Bakre MM, et al. Clinical validation of an immunohistochemistry-based CanAssist-Breast test for distant recurrence prediction in hormone receptor-positive breast cancer patients. Can Med. 2019; Epub ahead of print. 10.1002/cam4.2049 PMC6488210 30848103

[21] Nielsen T, Wallden B, Schaper C, Ferree S (2014). Analytical validation of the PAM50-based Prosigna breast Cancer prognostic gene signature assay and nCounter analysis system using formalin-fixed paraffin-embedded breast tumor specimens. BMC Cancer.

[22] Kronenwett R, Bohmann K, Prinzler J, Sinn BV (2012). Decentral gene expression analysis: analytical validation of the Endopredict genomic multianalyte breast cancer prognosis test. BMC Cancer.

[23] Robert AA, Floore A, Curry B (2007). Robust interlaboratory reproducibility of a gene expression signature measurement consistent with the needs of a new generation of diagnostic tools. BMC Genomics.

