# Supplementary material for: Analytical validation of CanAssist-Breast: an immunohistochemistry based prognostic test for hormone receptor positive breast cancer patients
Source: BMC Cancer. 2019 Mar 20;19:249. doi: 10.1186/s12885-019-5443-5 (PMC6425559; doi:10.1186/s12885-019-5443-5)
Supplement: Supplementary file 1 — Additional information on methods. (DOCX 14 kb) [file 12885_2019_5443_MOESM1_ESM.docx]

Title of the manuscript: **Analytical validation of CanAssist-Breast: An Immunohistochemistry based prognostic test**”

**Additional Methods:**

IHC staining details:

IHC staining is performed as follows. FFPE tissues are sectioned into 3-5µ slices using a Leica microtome (#RM2125RTS). Poly-L-Lysine coated slides (Pathnsitu) were used for taking sections. The sections are fixed on glass slides by placing them in a hot-air oven (Apollo Scientific) at 55°C for 1 hour. The slides are then de-paraffinized with xylene (Fisher Scientific) solution twice for 15 minutes each. Slides are rehydrated by washing twice with 100% alcohol for 5 minutes followed by 2 washes with 70% alcohol for 5 minutes, and finally with demineralized water (Nice Cat # D1505) for 5 minutes. Antigen retrieval is performed for each antibody as per the manufacturer’s instructions. Following antigen retrieval, slides are cooled completely to room temperature in the same buffer. On attaining room temperature the slides are washed in demineralized water for 5 minutes. After wiping extra moisture on the slide with a tissue, the tumor section is marked with a PAP pen. The rest of the steps are performed using the Novolink Polymer Secondary Kit (Leica, RE-7280K). Peroxidase block is added to each slide and incubated for 5 minutes. Slides are washed with wash buffer (10mM TBS-Tween 20, pH 7.4) twice, for 5 minutes each. After washing, the protein block is added and slides are incubated for 5 minutes. Slides are washed with wash buffer twice, for 5 minutes each. Post this appropriate antibody anti-CD44 (Thermo Scientific, Cat. # MA513890), anti-ABCC4 (Abnova, Cat. # H00010257-M03), anti-ABCC11 (Novus Biologicals, Cat. # NBP1-82623), anti-N-Cadherin (AbCam, Cat. # ab98952), anti-Cadherin-pan (Spring Biosciences, Cat. # E2364) is added to each slide. Dilution of primary antibody is performed as per the manufacturer’s instructions for all antibodies. All antibodies are obtained from commercial vendors. - Slides are incubated for 1 hour in a humidifying chamber with antibody. After primary antibody incubation, slides are washed with wash buffer twice, for 5 minutes each. Post-primary solution is added to the slides, and incubated for 30 minutes, followed by 2 washes with wash buffer as described previously. Following this, slides are incubated with Polymer for 30 minutes and then washed twice with wash buffer. Peroxidase activity is developed using DAB working solution for 5 minutes, following which the slides are rinsed with demineralized water for 2 minutes. Sections are counterstained with Hematoxylin (Fisher Scientific) for 8 minutes and rinsed in demineralized water for 8 minutes. The slides are subsequently dehydrated with 70%, 95% and 100% alcohol, each for 5 minutes. They are dried at room temperature and then incubated in Xylene for 5 minutes. Slides are dried and mounted with D.P.X. Mountant (NICE, Product # D30475).
