# Supplementary figures and images for: Analytical validation of CanAssist-Breast: an immunohistochemistry based prognostic test for hormone receptor positive breast cancer patients
Source: BMC Cancer. 2019 Mar 20;19:249. doi: 10.1186/s12885-019-5443-5 (PMC6425559; doi:10.1186/s12885-019-5443-5)

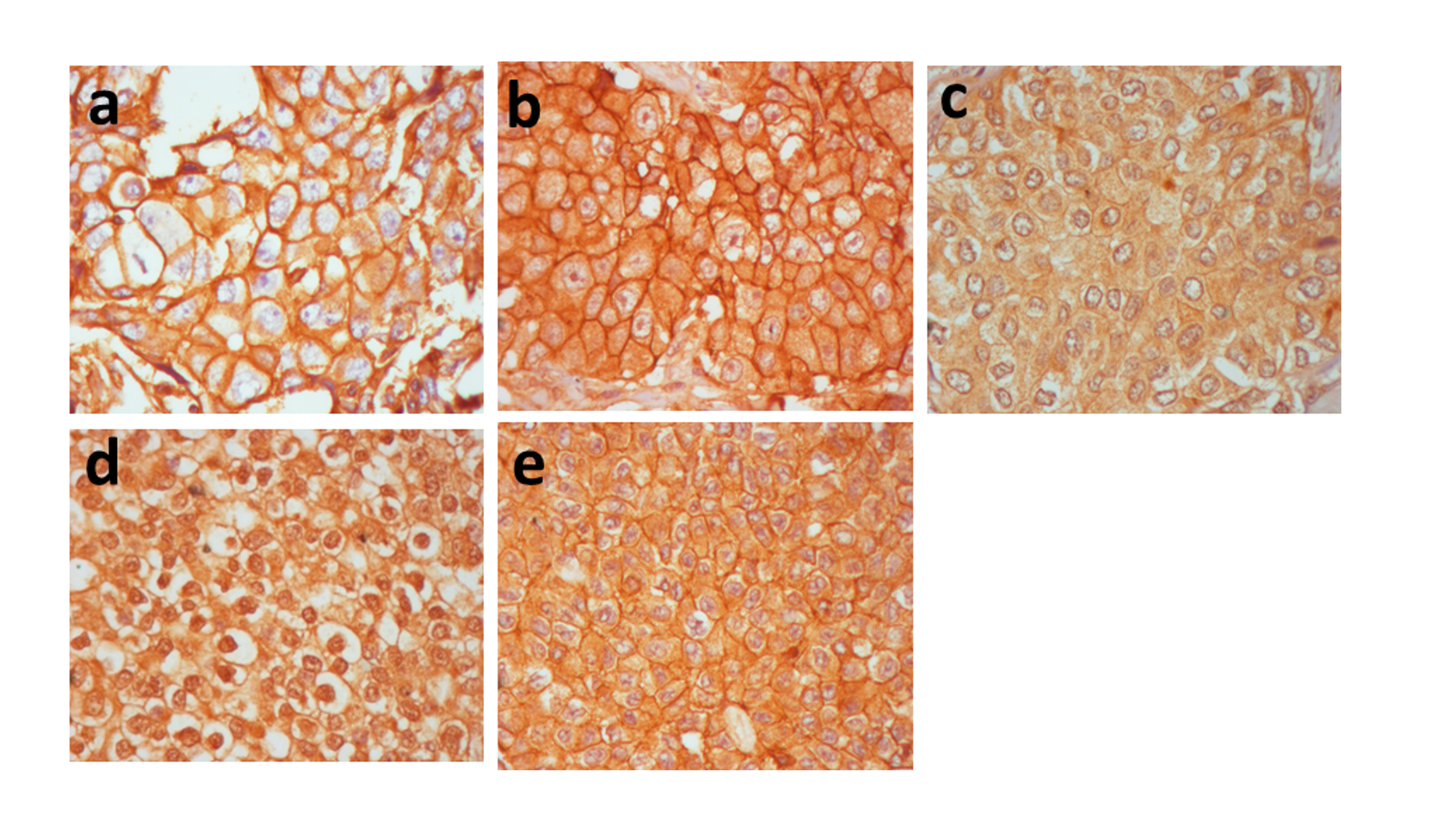

Supplement: Supplementary file 2 — Figure S1. Representative IHC images of the CanAssist-Breast biomarkers: IHC images of CanAssist-Breast biomarkers captured at 40X magnification a. CD44, b. ABCC4, c. ABCC11, d. N-cadherin, e. Pan-cadherin respectively. (TIF 10398 kb) [file 12885_2019_5443_MOESM2_ESM.tif]

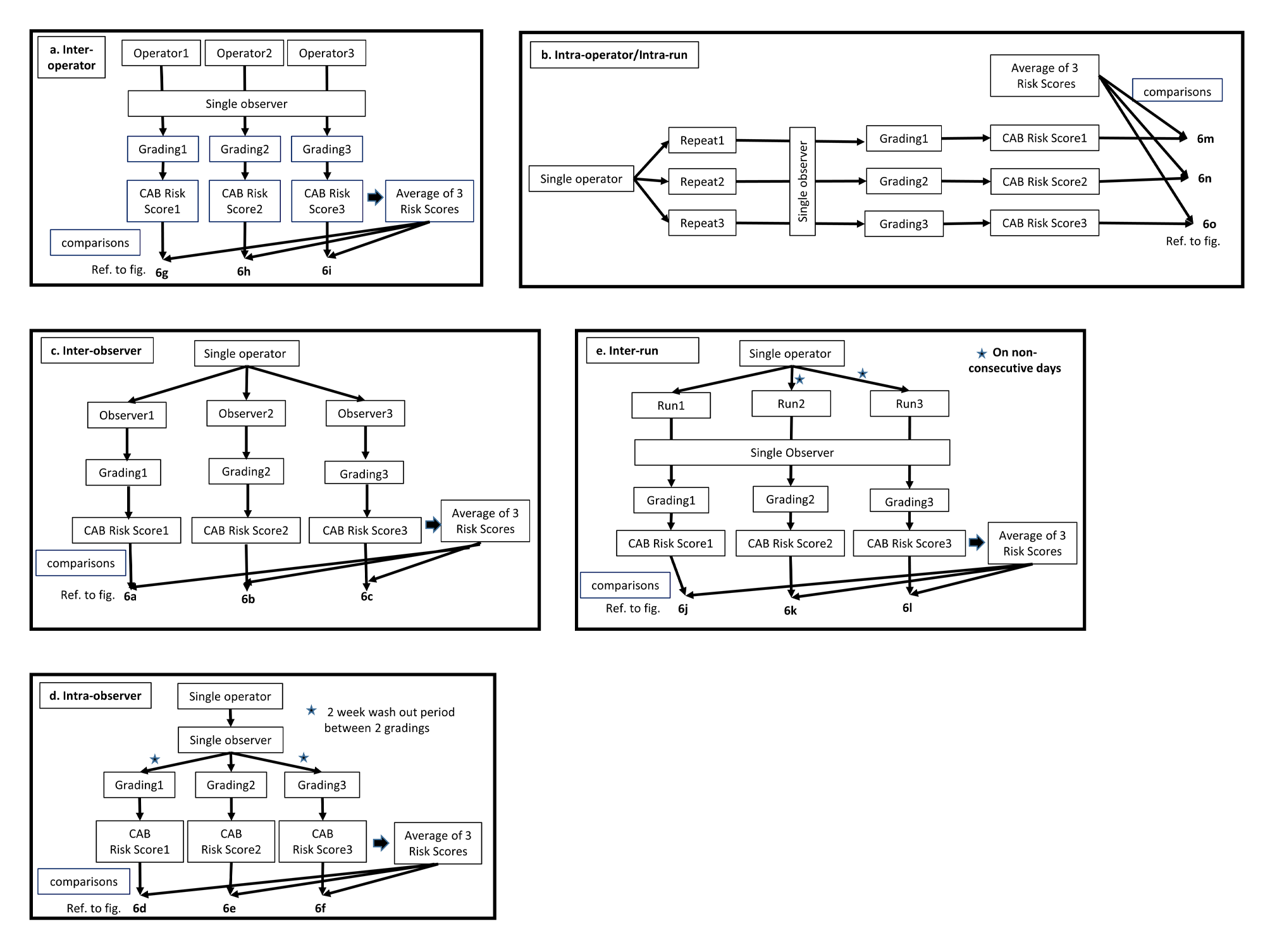

Supplement: Supplementary file 3 — Figure S2. Analysis employed for assessing ‘Precision on risk scores’. Schematic representation of the analysis employed in assessing precision on risk scores for all variables tested (a-e). (TIF 347 kb) [file 12885_2019_5443_MOESM3_ESM.tif]
